# Association between varicocele and hemorrhoidal disease in men presenting with groin pain: a retrospective comparative study

**DOI:** 10.1186/s12894-026-02145-x

**Published:** 2026-04-15

**Authors:** Sezgin Yeni, Mete Kilciler

**Affiliations:** 1 Mudanya University, Vocational School, Bursa, Turkey; 2 Vm Medical Park Bursa Hospital, Urology, Bursa, Turkey

**Keywords:** Constipation, Groin pain, Hemorrhoidal disease, Varicocele, Venous insufficiency

## Abstract

**Objective:**

To evaluate the independent association between varicocele and hemorrhoidal disease in men presenting with groin pain. This study considers varicocele not merely as a localized scrotal condition, but as a potential manifestation of systemic venous insufficiency, sharing common pathophysiological mechanisms with hemorrhoidal disease, such as venous dilation and elevated pressure.

**Materials and methods:**

Between January 2023 and September 2025, 292 men presenting with groin pain were assessed, and 186 met the inclusion criteria. Group 1 consisted of 81 men with varicocele who underwent varicocelectomy, and Group 2 included 105 men without varicocele (controls). Beyond univariate comparisons, a multivariable logistic regression model was utilized to identify independent predictors and adjust for potential confounders, including constipation. Model stability and calibration were verified using the Hosmer-Lemeshow test and Variance Inflation Factor (VIF) analysis.

**Results:**

There were no significant differences between groups regarding age, height, weight, body mass index, or smoking status (*p* > 0.05). Hemorrhoidal disease was significantly more frequent in the varicocele group than in controls (43.2% vs. 18.0%, *p* = 0.016). Multivariable logistic regression revealed that hemorrhoidal disease is a strong independent predictor of varicocele presence (Adjusted OR: 3.27; 95% CI: 1.66–6.59; *p* < 0.001). Additionally, constipation was identified as a significant independent predictor (Adjusted OR: 8.39; 95% CI: 2.61–37.64; *p* = 0.001). The model demonstrated high reliability with no multicollinearity (VIF = 1.00) and an acceptable goodness-of-fit (*p* = 0.054).

**Conclusion:**

Hemorrhoidal disease and constipation are independently associated with varicocele, consistent with a potential link through systemic venous insufficiency. Recognizing this association may facilitate a more comprehensive clinical evaluation of patients presenting with these interconnected venous conditions.

## Introduction

Varicocele is a common condition characterized by abnormal dilatation of the pampiniform venous plexus and is frequently encountered in men presenting with scrotal or groin pain and infertility [1]. It is widely accepted that varicocele represents a manifestation of systemic venous insufficiency rather than an isolated scrotal pathology [2]. Previous studies have suggested that venous disorders such as lower extremity varicose veins may be associated with varicocele, demonstrating a potential link with generalized venous dysfunction [3].

Hemorrhoidal disease is another prevalent venous disorder resulting from dilation and displacement of the hemorrhoidal venous plexus [4]. Similar to varicocele, hemorrhoids are associated with increased venous pressure, venous valve insufficiency, and impaired venous drainage [5, 6]. Chronic constipation is frequently observed in patients with hemorrhoidal disease and may exacerbate venous dilation by promoting straining and transient elevations in intra-abdominal pressure [7]. Despite these shared pathophysiological mechanisms, the relationship between varicocele and hemorrhoidal disease has not been sufficiently investigated.

Understanding whether varicocele is associated with other venous disorders such as hemorrhoids may provide insight into its systemic nature and help clinicians adopt a more comprehensive approach to patient evaluation. Therefore, the aim of this study was to investigate the frequency of hemorrhoidal disease in patients with varicocele compared to patients without varicocele presenting with groin pain.

## Materials and methods

### Study design and ethical approval

This study was approved by the Mudanya University Health Sciences Ethics Committee (Reference No: E-40839601-50.04-183) and conducted in accordance with the principles of the Declaration of Helsinki (1975, revised 2008). Patient data were collected prospectively and analyzed retrospectively.

This study was designed as a retrospective observational comparative study conducted at a single tertiary care center.

Informed consent was obtained from all individual participants included in the study.

### Patient selection

Between January 2023 and September 2025, a total of 292 male patients presented to the urology outpatient clinic with complaints of groin pain. Following physical examination and scrotal Doppler ultrasonography, 117 patients were diagnosed with varicocele, while 175 patients were found to have no varicocele.

Among these patients, those who attended follow-up visits, had available medical records regarding hemorrhoidal disease, and provided verbal confirmation were included in the study.

After applying the exclusion criteria (*n* = 106), a total of 186 patients were included in the final analysis.

**Fig. 1:**
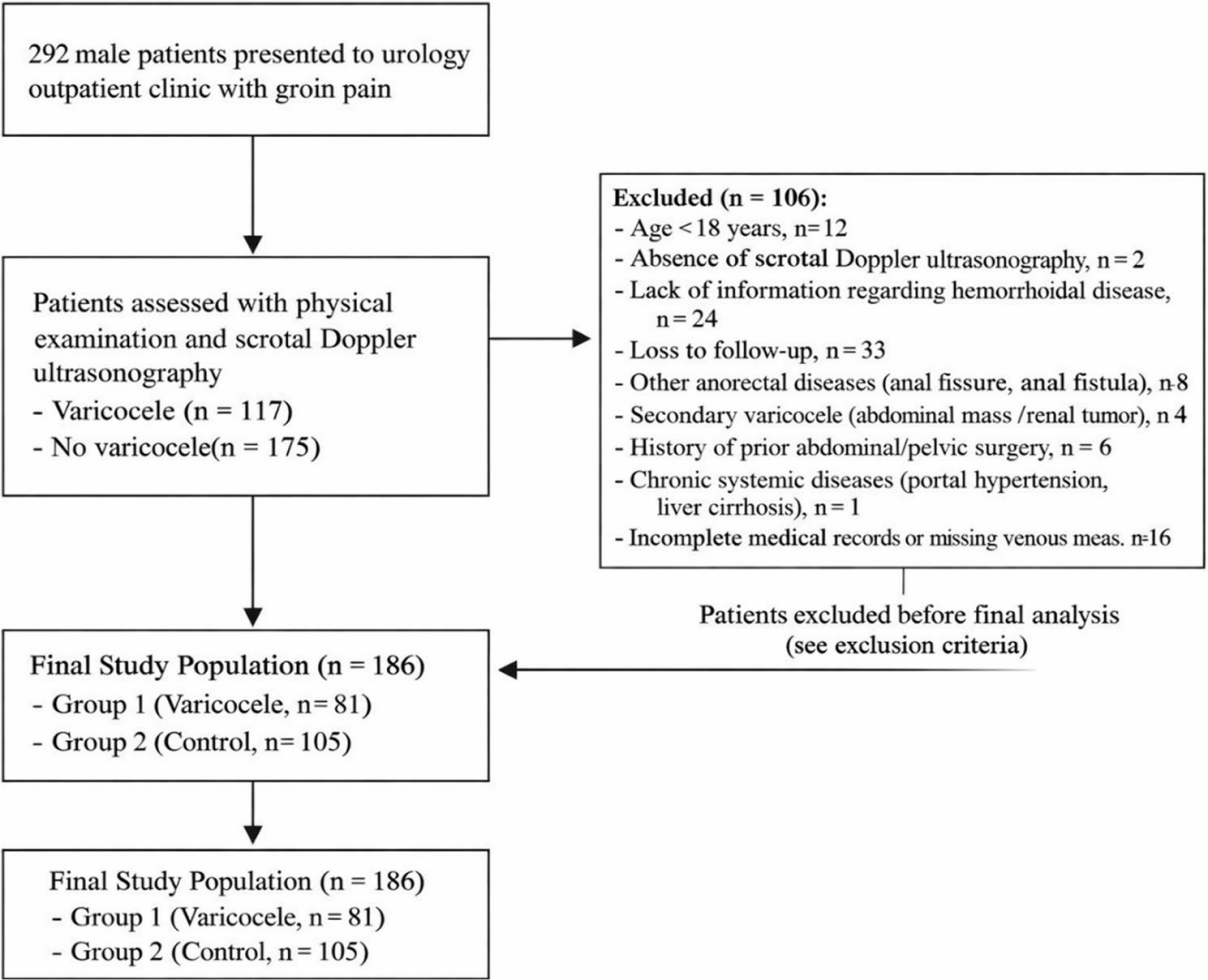
Flow diagram illustrating patient screening, exclusion criteria, and final study population

The patient selection process, including all inclusion and exclusion steps, is summarized in a STROBE-style flow diagram (Fig. 1).

### Exclusion criteria

Patients were excluded from the study if they met any of the following criteria:

- Age younger than 18 years (*n* = 12)
- Absence of scrotal Doppler ultrasonography (*n* = 2)
- Lack of information regarding hemorrhoidal disease (*n* = 24)
- Loss to follow-up (*n* = 33)
- Presence of other anorectal diseases such as anal fissure or anal fistula (*n* = 8)
- Secondary varicocele (abdominal mass/renal tumor) (*n* = 4)
- History of prior abdominal or pelvic surgery (*n* = 6)
- Chronic systemic diseases (portal hypertension, liver cirrhosis) (*n* = 1)
- Incomplete medical records or missing venous measurements (*n* = 16)

### Total excluded: *n* = 106

Patients were divided into two groups:

- Group 1 (*n* = 81): Patients diagnosed with varicocele based on physical examination and scrotal Doppler ultrasonography who subsequently underwent varicocelectomy.
- Group 2 (*n* = 105): Patients without evidence of varicocele, serving as the control group.

Laterality or bilaterality of varicocele was not separately analyzed, as the primary objective of the study was to evaluate the overall association between the presence of varicocele and hemorrhoidal disease.

To minimize potential selection bias, baseline demographic characteristics (age, BMI, and smoking status) were compared between groups.

### Hemorrhoidal disease assessment

The presence of hemorrhoidal disease was evaluated by questioning patients about hematochezia and reviewing medical records. A diagnosis of hemorrhoidal disease was confirmed if patients had previously been examined by a specialist physician and diagnosed with hemorrhoidal disease, and had received treatment from a general surgery or gastroenterology specialist, based on hospital records and patient self-report.

Hemorrhoidal disease was identified based on medical records, prior specialist evaluation, and patient-reported history. No standardized proctologic examination or validated staging system was applied.

A standardized proctologic examination or hemorrhoidal disease staging was not performed at the time of the study, as this retrospective approach was intended to reflect routine clinical practice and avoid additional invasive procedures.

Varicocele diagnosis was based on physical examination and/or scrotal Doppler ultrasonography.

Constipation status was determined based on patient-reported symptoms, including straining, hard stools, or decreased bowel frequency, as documented in medical records, rather than standardized diagnostic criteria such as the Rome IV classification [8].

Constipation was included as a key potential confounder in the analysis.

### Statistical analysis

Statistical analyses were performed using IBM SPSS Statistics for Windows, Version 26.0 (IBM Corp., Armonk, NY, USA). Continuous variables were expressed as median and interquartile range (IQR) or as mean ± standard deviation, depending on the distribution’s normality, assessed by the Shapiro-Wilk test. Categorical variables were presented as frequencies and percentages.

Differences between groups were analyzed using the independent samples t-test or the Mann–Whitney U test for continuous variables, as appropriate. The association between categorical variables was evaluated using the Chi-square test. To determine the magnitude of clinical associations for categorical data, the phi (ɸ) coefficient was calculated as an effect size measure. Additionally, effect sizes (r) were calculated for continuous variables. All effect size values were interpreted according to Cohen’s established criteria.

To identify independent predictors of varicocele presence, a multivariable logistic regression analysis was conducted. Variables with *p* < 0.25 in the univariate analysis were included in the multivariable model using the “enter” method.

Variables were selected based on both clinical relevance and univariate statistical significance.

Constipation was included in the multivariable model as a primary confounding variable.

The stability and calibration of the final model were assessed using the Omnibus test for overall model significance and the Hosmer–Lemeshow test for goodness-of-fit. Furthermore, multicollinearity among independent variables was rigorously screened using the Variance Inflation Factor (VIF).

Results of the regression analysis are presented as Odds Ratios (OR) with 95% Confidence Intervals (CI).

A *p*-value < 0.05 was considered statistically significant for all analyses.

## Results

The baseline demographic and clinical characteristics of the study population are presented in Table 1. No statistically significant differences were observed between the varicocele group (*n* = 81) and the control group (*n* = 105) regarding age, height, weight, body mass index (BMI), or smoking status (*p* > 0.05 for all).

**Table 1:** Baseline demographic characteristics of the study groups

Variable	Varicocele Group (*n* = 81)	Control Group (*n* = 105)	*p*-value
Age (year)	35 (29–38)	34 (30–36)	0.831^a^
Height (cm)	177 (172–182)	177 (173–180)	0.750^a^
Weight (kg)	81.8 ± 11.6	81.9 ± 11.3	0.959^b^
BMI (kg/m²)	26.2 ± 3.5	26.3 ± 3.9	0.786^b^
Smoking, n (%)	47 (58%)	58 (55.2%)	0.817^c^

Data are presented as mean ± standard deviation for normally distributed variables, median (interquartile range: Q1–Q3) for non-normally distributed variables, and n (%) for categorical variable

*BMI* Body mass index

a: Mann-Whitney U test

b: Independent samples t-test

c: Chi-square test

The median age was 35.0 (IQR: 29.0–38.0) years in the varicocele group and 34.0 (IQR: 30.0–36.0) years in the control group (*p* = 0.831). There were no significant differences in anthropometric measurements, including mean weight (81.8 ± 11.6 kg vs. 81.9 ± 11.3 kg; *p* = 0.959) and mean BMI (26.2 ± 3.5 kg/m2 vs. 26.3 ± 3.9 kg/m2; *p* = 0.786). Smoking prevalence was 58.0% (*n* = 47) in the varicocele group and 55.2% (*n* = 58) in the control group, showing no statistical difference between the cohorts (*p* = 0.817). 

The clinical characteristics and venous measurements of the study groups are presented in Table 2. The median venous diameter was significantly larger in the varicocele group compared to the control group (3.70 [IQR: 3.30–4.00] mm vs. 1.90 [IQR: 1.80–1.90] mm; *p* < 0.001). This difference demonstrated a large effect size (*r* = 0.88), highlighting a pronounced venous distension in the varicocele cohort.

**Table 2:** Clinical and venous characteristics of patients with and without varicocele

Variable	Varicocele Group (*n* = 81)	Control Group (*n* = 105)	*p*-value
Constipation, n (%)	17 (21%)	3 (2.9%)	**< 0.001** ^**c**^
Venous diameter (mm)	3.70 (3.30–4)	1.90 (1.80–1.90)	**< 0.001** ^**a**^
Hemorrhoidal disease, n (%)	35 (43.2%)	19 (18.1%)	**< 0.001** ^**c**^

Data are presented as median (interquartile range: Q1–Q3) or n (%)

Values in bold represent statistically significant differences (p < 0.05)

a: Mann-Whitney U test

c: Chi-square test

Regarding the primary outcomes, hemorrhoidal disease was significantly more prevalent in patients with varicocele (43.2%, *n* = 35) than in those without (18.1%, *n* = 19; *p* < 0.001). This association showed a small-to-medium effect size (ɸ = 0.26), indicating a statistically significant clinical relationship between the two conditions.

Similarly, a significant difference in the prevalence of constipation was observed. Constipation was reported by 21.0% (*n* = 17) of the patients in the varicocele group, compared to 2.9% (*n* = 3) in the control group (*p* < 0.001). The analysis of this finding yielded a small-to-medium effect size (ɸ = 0.27), suggesting that constipation is a notable factor frequently associated with the varicocele group. 

To determine the independent risk factors associated with varicocele, the variables in Table 3 were primarily evaluated using univariate logistic regression. Following the univariate stage, variables with *p* < 0.25 were included in the multivariable logistic regression model. Among the variables examined in the univariate model, hemorrhoidal disease and constipation met the *p* < 0.25 criterion.

**Table 3:** Logistic regression analysis of risk factors associated with the presence of varicocele

Variables	Univariate Model		Multivariable Model	
	OR (95% CI)	*p*-value	OR (95% CI)	*p*-value
Age	1.00 (0.94–1.07)	0.963	—	—
BMI (kg/m2)	0.99 (0.91–1.07)	0.784	—	—
Smoking status (Ref: No)	1.12 (0.62–2.02)	0.704	—	—
Hemorrhoidal disease (Ref: No)	3.44 (1.79–6.79)	**< 0.001**	3.27 (1.66–6.59)	**< 0.001**
Constipation (Ref: No)	9.03 (2.89–39.79)	**0.001**	8.39 (2.61–37.64)	**0.001**

*OR* Odds Ratio, *CI* Confidence Interval, *BMI* Body Mass Index, *Ref* Reference category

Model Diagnostics for Multivariable Model: Omnibus Model Significance *p* < 0.001; Hosmer-Lemeshow Goodness of Fit *p* = 0.054

Variables with *p* < 0.25 in the univariate analysis were included in the multivariable model

The resulting multivariable model demonstrated high statistical significance (Omnibus test, *p* < 0.001) and was consistent with the data (Hosmer-Lemeshow test, *p* = 0.054). Furthermore, to ensure model stability, multicollinearity diagnostics were conducted among the independent variables. The Variance Inflation Factor (VIF) was 1.00 for both hemorrhoidal disease and constipation, indicating no multicollinearity and confirming the model’s structural integrity.

According to the multivariable analysis, the risk of varicocele was 3.27 times higher in patients with hemorrhoidal disease than in those without (95% CI: 1.66–6.59; *p* < 0.001). Furthermore, constipation was identified as a strong independent predictor; patients with constipation had an 8.39-fold increased risk of varicocele compared with those without constipation (95% CI: 2.61–37.64; *p* = 0.001).

## Discussion

In the present study, we observed a higher prevalence of hemorrhoidal disease in patients with varicocele compared to those without (43.2% vs. 18.0%, *p* = 0.016). However, given the observational design of the study, this finding should be interpreted as an association rather than a causal relationship. After adjustment for potential confounders, including constipation, hemorrhoidal disease remained an independent predictor of varicocele (Adjusted OR: 3.27; 95% CI: 1.66–6.59; *p* < 0.001), suggesting that shared risk factors such as increased intra-abdominal pressure may partially explain this association. This finding is consistent with the hypothesis that varicocele may be part of a systemic venous disorder rather than an isolated local pathology.

Age, height, weight, body mass index, and smoking status were comparable between the two groups, suggesting that these variables were unlikely to have confounded the observed association between varicocele and hemorrhoidal disease [9]. As expected, venous diameter and constipation were significantly higher in the varicocele group, confirming the accuracy of patient classification [10, 11]. Although some studies have reported a positive association between increasing body mass index and venous insufficiency, other investigations have demonstrated that a higher body mass index does not necessarily correlate with an increased prevalence of varicocele [12–14]. In line with these findings, the lack of significant differences in body mass index and smoking status between groups in our study suggests that the observed association between varicocele, hemorrhoidal disease, and constipation may be independent of common lifestyle-related risk factors.

Constipation is a prevalent and frequently chronic gastrointestinal motility disorder characterized by symptoms such as excessive straining, hard stool consistency, and reduced bowel movement frequency [15]. Persistent constipation is frequently observed in patients with varicocele and hemorrhoidal disease and may be associated with transient elevations in intra-abdominal pressure. In addition, previous studies have demonstrated that constipation is commonly associated with the presence and progression of hemorrhoidal disease [7, 16].

Consistent with these findings, our study revealed a significantly higher prevalence of hemorrhoidal disease among patients with varicocele compared to the control group, which may be partially explained by the increased occurrence of constipation in the varicocele group. Constipation was associated with varicocele and hemorrhoids.

In our multivariable analysis, hemorrhoidal disease was confirmed as an independent predictor of varicocele after adjusting for constipation and other covariates, addressing potential confounding.

In multivariable logistic regression, the odds of hemorrhoidal disease being greater in varicocele patients was 3.27 times when adjusted for constipation and other covariates. Diagnostic assessments confirmed the absence of multicollinearity (VIF = 1.00) and acceptable model fit (Hosmer-Lemeshow *p* = 0.054, Omnibus *p* < 0.001), supporting the robustness of the findings.

The relationships between varicocele and hemorrhoidal disease and bowel habits were shown to differ and are not entirely explained by the mechanisms of increased intra-abdominal pressure and chronic straining attributed to constipation.

The underlying mechanism linking varicocele and hemorrhoidal disease may involve generalized venous insufficiency, increased intra-abdominal pressure, or congenital weakness of venous walls and valves [17]. To assess the robustness of the multivariable model results, diagnostic tests were performed. There was no collinearity between hemorrhoidal disease and constipation, so the variables entered into the model independently. The overall model was also highly significant. These results are consistent with the ‘systemic venous insufficiency’ hypothesis with the possibility of a primary venodilatory state predisposing to venous dilatation in the pampiniform and haemorrhoidal plexuses, while acknowledging that residual confounding cannot be entirely excluded due to the observational study design.

Both conditions share similar hemodynamic disturbances, including venous reflux and elevated venous pressure, which may predispose susceptible individuals to multiple venous pathologies [18].

Hemorrhoidal disease should not be regarded solely as a venous disorder; in advanced stages, it may impair defecatory mechanics by causing functional outlet obstruction and repetitive straining [19]. This altered defecation dynamics can result in sustained elevations of intra-abdominal pressure over time [20]. Chronic increases in intra-abdominal pressure may be associated with impaired venous return from the testicular veins, thereby being linked to varicocele, particularly in individuals with underlying venous valve insufficiency [21]. Although this proposed mechanism remains hypothetical, it offers a plausible pathophysiological link between hemorrhoidal disease and varicocele and warrants further investigation in prospective studies incorporating standardized functional anorectal and hemodynamic assessments.

The potential impact of varicocelectomy on hemorrhoidal disease symptoms was not evaluated in the present study, as postoperative proctologic follow-up data were not routinely available; however, changes in venous hemodynamics following varicocelectomy may be associated with changes in hemorrhoidal symptoms.

Although a few studies have investigated the relationship between varicocele and hemorrhoidal disease, the existing evidence remains limited and inconclusive. Our findings, derived from a rigorously defined cohort with multivariable adjustment, add to the literature by further highlighting hemorrhoids as a potential manifestation of systemic venous pathology in patients with varicocele, while emphasizing that these results reflect association rather than causation.

### Study limitations

Our study has several limitations that warrant consideration. First, although patient data were collected prospectively, the analysis is retrospective and observational, which inherently precludes establishing a definitive causal relationship between varicocele and hemorrhoidal disease. The retrospective nature of the analysis introduces a risk of selection bias. While a strong independent association was identified, our findings should be interpreted as a clinical correlation rather than a causal relationship. The inclusion of patients presenting with groin pain and the restriction of the varicocele group to those undergoing surgery may further limit the generalizability of the findings.

Second, our study population represents a highly selected symptomatic cohort and surgical subgroup at a single tertiary center. This may introduce selection bias and limit the generalizability (external validity) of our results to the broader asymptomatic population. However, to mitigate this risk, we applied strict inclusion and exclusion criteria and ensured that baseline demographic characteristics (Age, BMI, Smoking) were statistically homogeneous across groups, as shown in Table 1, thereby reducing the influence of major confounding factors. Although multivariable logistic regression analysis was performed in the revised analysis, residual confounding cannot be excluded, particularly given the relatively limited sample size.

Third, the ascertainment and staging of hemorrhoidal disease were based on medical records and clinical evaluations in a surgical setting rather than a purely objective, standardized screening protocol. Similarly, constipation was assessed based on patient-reported symptoms rather than validated criteria such as the Rome IV classification, which may introduce a degree of subjectivity and potential misclassification bias.

Fourth, while constipation was adjusted for as a primary confounder in our multivariable model, other unmeasured lifestyle factors—such as dietary fiber intake or physical activity levels—could not be evaluated. Nevertheless, the independent association observed after adjusting for constipation suggests a statistically robust association between the two conditions.

Finally, while the single-center design may limit the generalizability of the findings, the consistent, homogeneous evaluation of all patients by the same clinical team minimizes interobserver variability and strengthens the study’s internal reliability. Despite these limitations, achieving an adequate Events Per Variable (EPV) ratio in our regression model and the absence of multicollinearity (VIF = 1.00) support the statistical robustness of the reported independent association. Therefore, the findings should be interpreted with caution, and prospective studies with standardized assessments are needed.

## Conclusion

Our study demonstrates a significantly higher prevalence of hemorrhoidal disease and constipation in patients with varicocele compared to those without varicocele. However, given the observational design, this association may be influenced by shared risk factors such as constipation and increased intra-abdominal pressure, and should be interpreted as an association rather than a causal relationship. These findings support the concept that varicocele may be linked to systemic venous insufficiency and that increased intra-abdominal pressure related to constipation may be associated with the coexistence of these conditions. Clinicians should be aware of these associations and consider evaluating patients with varicocele for other venous disorders, including hemorrhoidal disease, as well as assessing bowel habits. Further prospective studies are required to better clarify the nature and underlying mechanisms of these relationships.

## Data Availability

The datasets used and/or analysed during the current study are available from the corresponding author on reasonable request.

## Abbreviations

BMI: Body Mass Index
CI: Confidence Interval
EPV: Events Per Variable
IQR: Interquartile Range
OR: Odds Ratio
VIF: Variance Inflation Factor
